# AMS 3.0: prediction of post-translational modifications

**DOI:** 10.1186/1471-2105-11-210

**Published:** 2010-04-28

**Authors:** Subhadip Basu, Dariusz Plewczynski

**Affiliations:** 1 Department of Computer Science and Engineering, Jadavpur University, Kolkata - 700032, India; 2 Interdisciplinary Centre for Mathematical and Computational Modeling, University of Warsaw, 5a Street, 02-106 Warsaw, Poland

## Abstract

**Background:**

We present here the recent update of AMS algorithm for identification of post-translational modification (PTM) sites in proteins based only on sequence information, using artificial neural network (ANN) method. The query protein sequence is dissected into overlapping short sequence segments. Ten different physicochemical features describe each amino acid; therefore nine residues long segment is represented as a point in a 90 dimensional space. The database of sequence segments with confirmed by experiments post-translational modification sites are used for training a set of ANNs.

**Results:**

The efficiency of the classification for each type of modification and the prediction power of the method is estimated here using recall (sensitivity), precision values, the area under receiver operating characteristic (ROC) curves and leave-one-out tests (LOOCV). The significant differences in the performance for differently optimized neural networks are observed, yet the AMS 3.0 tool integrates those heterogeneous classification schemes into the single consensus scheme, and it is able to boost the precision and recall values independent of a PTM type in comparison with the currently available state-of-the art methods.

**Conclusions:**

The standalone version of AMS 3.0 presents an efficient way to indentify post-translational modifications for whole proteomes. The training datasets, precompiled binaries for AMS 3.0 tool and the source code are available at http://code.google.com/p/automotifserver under the Apache 2.0 license scheme.

## Background

Post-translational modification (PTM) is the chemical modification of a protein after its translation. During protein synthesis, a protein is build using twenty different amino acids, yet after translation a post-translational modification of amino acids can be observed by attaching to them other biochemical functional groups such as acetate, phosphate, various lipids and carbohydrates, by changing the chemical nature of an amino acid, or by making structural changes, like the formation of disulfide bridges. In the advent of massive (complex and time-consuming) sequencing experiments, the availability of whole proteomes requires accurate computational techniques for investigation of protein modification sites for the high-throughput scale. To address these needs we propose here our improved technique to identify PTM sites by using artificial neural network, trained on proteins from the current version of Swiss-Prot database [1] and Phospho.ELM dataset [2,3].

The automatic prediction of PTM sites is now very important area of interest for the bioinformatics research community. The currently available PTM prediction tools can be categorized into four major groups based on the used types of classification schemes. The first category includes general PTM related resources like ELM [4] that perform rapid regular expression pattern search in order to predict Eukaryotic Linear Motifs (ELMs) in protein sequences. Another web service, namely PROSITE [5] predicts many types of PTMs based on the consensus of sequence patterns. Consensus based approaches combine several signature recognition methods to scan a given query protein sequence against observed protein signatures. The Scansite tool [6] predicts kinase-specific and signal transduction relevant motifs. The conserved sequence motifs represent footprints of important biochemical properties or biological functions performed by those proteins. GPS approach predicts kinase-specific phosphorylation sites from protein primary sequences for 71 different PK groups by family-based phosphorylation scoring technique [7,8]. The PHOSITE [9] algorithm for prediction of phosphorylation sites is based on case-based sequence analysis to obtain predictions with constant specificity and sensitivity.

The second class of methods covers different neural network prediction tools. These include phosphorylation related prediction servers like NetPhos [10] and NetPhosK [11,12], glycosylation based tools like NetOGlyc [13], NetNGlyc, DictyOGlyc [14], YinOYang [15], prediction of cleavage sites in protein sequences [16-18], prediction of N-terminal myristoylation on protein sequences [19] and many others. The most popular among these servers is NetPhosK that allows the user to choice its preferred 'threshold' value during prediction. In our manuscript we present the results with three threshold levels: 0.3, 0.5 and 0.7 for making the predictions and they are called respectively as NetPhosK_0.3, NetPhosK_0.5 and NetPhosK_0.7.

The third category of methods involves several support vector machine (SVM) based prediction techniques. Among the recent works, protein methylation site prediction is attempted using bayesian feature extraction technique and SVM based classifier [20]. In another work, the prediction of Lysine acytelation sites is done using SVM [21] based classifiers. PredPhospho [22] is another SVM based system that attempts to predict phosphorylation sites and the type of kinase that acts at each site. Our previously developed web-server AutoMotifServer (AMS) [23] for prediction of post-translational modification sites in protein sequences, also uses SVM classifier with both linear and polynomial kernels. This software is available freely at http://ams2.bioinfo.pl/. KinasePhos 2.0 [24] is another web server for identifying protein kinase-specific phosphorylation sites based on 9 amino acid long sequences and coupling patterns. There are several options provided in this prediction program, including three different levels of specificity: 90%, 95% and 100%. These three options are named respectively KinasePhos_90, KinasePhos_95 and KinasePhos_100.

The fourth category consists from the remaining other types of machine learning based PTM prediction tools. It includes for example Sulfinator [25] that predicts tyrosine sulfation sites using a combination of HMM models; prediction of glycosylation sites using random forests [26]; PPSP prediction of PK-specific phosphorylation sites [8] that deploys Bayesian decision theory (BDT); and many others. The PPSP predicted the plausible phosphorylation sites accurately for approximately 70 PK (Protein Kinase) groups. For our tests we choose the PPSP_balanced model that seems to provide the best overall performance for all types of protein families. In the important work of Wan et al. [27] the efficient meta predictor is designed that organize and process predictions from individual source prediction algorithms. They compiled and evaluated their technique on four unbiased phosphorylation site datasets, namely the four major protein kinase families: CDK, CK2, PKA and PKC. In addition to the aforementioned classification software/tools, the dbPTM database [28] compiles diverse information on protein post-translational modifications (PTMs), such as the catalytic sites, solvent accessibility of amino acid residues, protein secondary and tertiary structures, protein domains and protein variations. The database includes a majority of the experimentally validated PTM sites from Swiss-Prot, PhosphoELM and O-GLYCBASE. The recent survey [29] describes available resources for predicting kinase-specific phosphorylation sites from sequence properties. They compare strengths and weaknesses of variety of prediction tools, as described above.

Despite almost ten years of work and above reported computational solutions, still we are unable to boost the precision of *in silico *methods to be really useful in high throughput context of personalized medicine. Therefore, the present research improvements concentrate on two important factors. The first is to further optimize prediction accuracy in comparison with the current state-of-the-art methods for variety of PTM sites, especially focusing on selecting more informative feature descriptors. The second factor is the speed of a prediction procedure that is needed for the virtual screening of whole proteomes. Therefore, we present here an extensively optimization scheme for selecting the most informative amino acids features, than used for training the very fast machine learning method, namely artificial neural network.

We have improved significantly the accuracy of the previous versions of AMS prediction tool [23,30] using an efficiently designed Multi Layer Perceptron (MLP) pattern classifier. In the current version of the tool, the query protein sequences are dissected into overlapping short segments. Ten different physico-chemical features represent each amino acid from a sequence segment; therefore the nine amino acids segment is represented as the point in a 90 dimensional abstract space of protein characteristics. The MLP used in this work, special Artificial Neural Network (ANN) algorithm, is developed to replicate *learning *and *generalization *abilities of human's behavior with an attempt to model the functions of *biological neural networks *of the human brain. The MLP architecture is build from a feed-forward layered network of *artificial neurons*, where each artificial neuron in the MLP computes a *sigmoid function *of the weighted sum of all its inputs.

**Figure 1:**
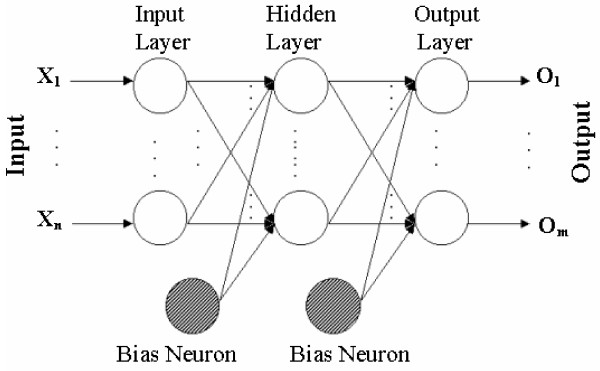
**MLP algorithm**. A block diagram of an MLP shown as a feed forward layered neural network.

The MLP based ANNs (see Figure 1) are observed to be capable of classifying highly complex and nonlinear biological sequence patterns, where correlations between amino acid positions are important. Unlike earlier attempts, in the current design of the neural network we have implemented three different network models for each of the PTM types, by independently optimizing the network weights for three factors: optimum recall (sensitivity); precision; and maximizing the receiver operating characteristics of the prediction model. The consensus build by those three ANNs for each type of PTM gives an additional advantage in comparison to the previously reported ANN based PTM prediction models. In our previous publications on AMS [23,30], the SVM based classification model failed to classify several PTM types with limited number of positive samples. The current MLP based design is much better suited to highly unbalanced ratio between positives and negatives in the training dataset, therefore preferred over the previously chosen SVM based approach.

Summarizing, the AMS 3.0 tool (available at http://bio.icm.edu.pl/~darman/ams3 and http://code.google.com/p/automotifserver/) integrates heterogeneous classification schemes for different PTM types, and it is designed to boost both the efficiency and speed in comparison with previously presented computational methods. As discussed earlier, the neural network is designed in much more efficient and unique way to predict post-translational modifications than previously used ANN algorithms. A detailed discussion on the architecture of the neural network, details of the machine learning algorithm is given in the first section. The next section presents the results on several benchmarking datasets, and finally the discussion part of the paper report the major advances of our work.

## Results

The performance of the networks is evaluated on the training and test datasets for each of the PTM types. During training of the feed-forward neural network with back-propagation learning algorithm, the learning rate (η) and acceleration factors (δ) are chosen as 0.8 and 0.8 respectively. The classification performance is described by the following measures of accuracy:(1)

where, TP is the number of true positives, FP is the number of false positives, TN is the number of true negatives, and FN is the number of false negatives. The classification error (E) is used to provide an overall error measure, recall (R) corresponds to the percentage of correct predictions, precision (P) measures the percentage of observed positives that are correctly predicted, true positive rate (TPR) is similar to recall or sensitivity measure and false positive rate (FPR) estimates the false alarm rate or fall-out. We also estimate the receiver operating characteristic (ROC) by plotting the fraction of true positives (TPR) vs. the fraction of false positives (FPR). More specifically, the ROC curves are drawn for both training and test datasets in each of the three runs of experiment, i.e., optimal AUC, recall and precision values. This ROC analysis provides a tool to select possibly optimal network models and to discard suboptimal ones independently from the class distribution. The area under the ROC curve (AUC) is also calculated in the current experiment and presented in Table 1. The AUC is equivalent to the probability that a classifier will rank a randomly chosen positive instance higher than a randomly chosen negative one [31].

**Table 1:** Single Amino-Acids Features Selection.

Accession number	Brief feature description	Reference	Selected/Rejected
ARGP820101	Hydrophobicity index	Eur. J. Biochem. 128, 565-575 (1982)	Rejected
BIOV880101	Information value for accessibility; average fraction 35%	Protein Engineering 2, 185-191 (1988)	Selected
BIOV880102	Information value for accessibility; average fraction 23%	Protein Engineering 2, 185-191 (1988)	Selected
BLAS910101	Scaled side chain hydrophobicity values	Analytical Biochemistry 193, 72-82 (1991)	Selected
BLAS910101	Scaled side chain hydrophobicity values	Analytical Biochemistry 193, 72-82 (1991)	Rejected
BULH740101	Surface tension of amino acid solutions: A hydrophobicity scale of the amino acid residues	Arch. Biochem. Biophys. 161, 665-670 (1974)	Rejected
FASG760101	Molecular weight	Handbook of Biochemistry and Molecular Biology, 3rd ed., Proteins - Volume 1, CRC Press, Cleveland (1976)	Rejected
HOPA770101	Hydration number	Intermolecular Interactions and Biomolecular Organizations, Wiley, New York (1977)	Selected
KRIW710101	Side chain interaction parameter	Biochim. Biophys. Acta 229, 368-383 (1971)	Selected
KRIW790101	Side chain interaction parameter	Biochim. Biophys. Acta 576, 204-228 (1979)	Selected
KRIW790102	Fraction of site occupied by water	Biochim. Biophys. Acta 576, 204-228 (1979)	Selected
KRIW790103	Side chain volume	Biochim. Biophys. Acta 576, 204-228 (1979)	Selected
LAWE840101	Transfer free energy, CHP/water	J. Biol. Chem. 259, 2910-2912 (1984)	Selected
OOBM850105	Optimized side chain interaction parameter	Bull. Inst. Chem. Res., Kyoto Univ. 63, 82-94 (1985)	Selected
WARP780101	Average interactions per side chain atom	J. Mol. Biol. 118, 289-304 (1978) (Gly 0.81)	Rejected

List of experimentally chosen features used in the current experiment from the AAindex database with the corresponding feature accession number, brief description and the journal reference.

The detailed performance analysis of the current software using three random sub-sample validations on training and test datasets of different PTM types is given in the Additional file 1. The three sets of results (corresponding to the AUC, Recall and Precision optimizations) for each PTM type corresponds to the training of the network based on the optimized AUC area, recall and precision values for the random test dataset under consideration. Each such network is trained with possible variations of hidden neurons from 2 to 20 (with step size 2). The number of hidden neurons, for which a given network is observed to give best possible recognition accuracy, is also listed in the detailed experimental datasheet of the Additional file 1. It may be observed that the high recall value (more than 70%), reasonable precision (more than 50%) and AUC area (more than 0.75) is achieved on independent test datasets of most of the PTM types.

The performance of the current technique is observed to be significantly better for most PTM types in comparison with the results of the previous version of AutoMotif Server, i.e., AMS 2.0. In AMS 2.0, classification results using Support Vector Machine on the training datasets for respective PTM types are shown, which improved the earlier recognition performance of the earlier version of the server AMS 1.0 [23]. A relative comparison of the training set results of our technique with the corresponding results of the AMS 2.0 is shown in Table 2. Detailed results related to respective test set accuracies and due to variations in nomenclature, the current version of Swiss Prot dataset sometimes could not be matched precisely with all the PTM types, experimented in AMS2. However, a detailed experiment is also conducted with the old dataset to show the level of improvement for the current methodology. The results in both cases (with old and new datasets) clearly indicate that the developed software outperforms/improves the performance of the previous server.

**Table 2:** Improvement of the performance of the AutoMotifServer 3.0.

		AMS 2.0		AMS 3.0			AMS 3.0 (New SwissProt dataset)		
**PTM type**	**Agent**	**Recall**	**Precision**	**Recall**	**Precision**	**AUC**	**Recall**	**Precision**	**AUC**

3,4-dihydroxyproline	-	88,89	66,67	95,45	87,5	0,962273	97,37	97,37	0,985088
4-carboxyglutamate	-	90,56	95,93	96,27	99,65	0,980856	96,54	99,35	0,982076
4-hydroxyproline	-	65,59	85,31	86,88	83,94	0,905902	95,03	94,14	0,969559
5-hydroxylysine	-	12,79	84,62	97,3	38,92	0,845236	90,16	95,93	0,948272
Asymmetric dimethylarginine	-	84,62	82,09	94,55	94,55	0,965227	-	-	-
dihydroxyphenylalanine	-	29,41	76,92	86,17	86,17	0,914601	-	-	-
Glycine amide	-	84,38	90	97,92	97,92	0,977679	99,3	99,3	0,984574
Hydroxyproline	-	28,8	85,71	89,62	94,06	0,942113	94,62	99,19	0,972567
Leucine amide	-	98,97	97,96	98,1	99,36	0,989049	-	-	-
Methionine amide	-	100	93,75	100	93,75	0,666667	-	-	-
N-acetylglycine	-	79,17	90,48	97,83	97,83	0,985797	-	-	-
N-acetylmethionine	-	100	95,42	98,4	98,93	0,990593	-	-	-
N-acetylserine	-	98,24	99,64	98,22	99,4	0,990124	-	-	-
N-acetylthreonine	-	76,47	88,64	89,8	97,78	0,94648	-	-	-
N6-acetyllysine	-	12,76	74,32	94,3	93,23	0,953465	-	-	-
N6, N6, N6-trimethyllysine	-	7,5	60	85,71	97,5	0,926065	-	-	-
Omega-N-methylated arginine	-	0	0	78	58,21	0,82	-	-	-
Phenylalanine amide	-	98,59	93,33	99	99	0,986667	-	-	-
Phospho	PKA	63,64	77,78	90,72	86,27	0,939608	97,39	95,39	0,983802
Phospho	PKC	32,56	84,85	86,17	97,59	0,928851	94,78	99,18	0,973369
Phospho	autocatalysis	13,16	96,15	79,35	93,59	0,888392	95,96	99,15	0,97851
Phospho	CDC2	85,71	80,9	89,55	75,95	0,916095	93,73	92,36	0,963488
Phosphoserine	-	97,06	91,34	94,33	95,56	0,94975	96,87	98,02	0,912848
Phosphoserine	PKA	68,42	78,31	91,67	72,64	0,922083	97,66	98,43	0,987096
Phosphoserine	PKC	23,29	80,95	96,43	95,29	0,975476	94,93	98,94	0,973939
Phosphoserine	autocatalysis	10	85,71	75	90,7	0,868311	91,57	99,13	0,957295
Phosphoserine	CK2	29,73	70,97	88,14	59,77	0,882345	93,02	98,23	0,962999
Phosphothreonine	-	46,12	75,46	78,16	69,73	0,828495	85,2	97,4	0,905598
Phosphothreonine	autocatalysis	0	0	73,47	80	0,849347	94,96	99,82	0,974381
Phosphotyrosine	-	9,32	71,2	84,24	96,69	0,91775	91,75	98,97	0,952053
Phosphotyrosine	autocatalysis	1,41	12,5	92	92	0,951404	96,82	97,21	0,979045
Pyrrolidone carboxylic acid	-	60,31	97,11	91,58	97,92	0,955236	-	-	-
Sulfotyrosine	-	70,19	85,88	98,39	97,6	0,988929	99,48	98,47	0,990853
Valine amide	-	94,29	97,06	98,57	98,57	0,986607	-	-	-
Phospho.ELM	-	98	91,68	-	-	-	98,56	96,07	0,949397
Phospho.ELM	Abl	0	0	-	-	-	81,82	52,94	0,862032
Phospho.ELM	AMPK_group	6,25	100	-	-	-	100	72,22	0,975
Phospho.ELM	ATM	92,98	81,54	-	-	-	94,87	90,24	0,967439
Phospho.ELM	CaM-KIIalpha	41,67	88,24	-	-	-	100	77,42	0,981081
Phospho.ELM	CaM-KII_group	14,55	88,89	-	-	-	82,05	94,12	0,906796
Phospho.ELM	CDK1	41,73	63,04	-	-	-	96,94	62,91	0,94711
Phospho.ELM	CDK2	7,14	45,45	-	-	-	92,31	64,86	0,928627
Phospho.ELM	CDK_group	59,8	67,03	-	-	-	95,65	67,35	0,947785
Phospho.ELM	CK1_group	0	0	-	-	-	86,67	72,22	0,911594
Phospho.ELM	CK2 alpha	38,98	67,65	-	-	-	78,82	70,53	0,872036
Phospho.ELM	CK2_group	43,33	72,22	-	-	-	73,33	64,36	0,839759
Phospho.ELM	EGFR	0	0	-	-	-	73,17	78,95	0,852909
Phospho.ELM	Fyn	0	0	-	-	-	87,5	84,85	0,9275
Phospho.ELM	GRK_group	2,7	100	-	-	-	83,33	90,91	0,911404
Phospho.ELM	GSK-3beta	18,37	75	-	-	-	85,29	64,44	0,896282
Phospho.ELM	GSK-3_group	12,5	66,67	-	-	-	65,22	93,75	0,823128
Phospho.ELM	IGF1R	26,09	100	-	-	-	85	73,91	0,90625
Phospho.ELM	IKK_group	0	0	-	-	-	95,83	92	0,973761
Phospho.ELM	InsR	6,67	60	-	-	-	70,97	88	0,848289
Phospho.ELM	Lck	11,76	60	-	-	-	80,56	74,36	0,884596
Phospho.ELM	Lyn	0	0	-	-	-	66,67	84,62	0,825137
Phospho.ELM	MAPK1	45,88	67,24	-	-	-	88,98	55,85	0,898547
Phospho.ELM	MAPK14	4	22,22	-	-	-	82,35	77,78	0,896017
Phospho.ELM	MAPK3	74,7	78,48	-	-	-	87,93	77,27	0,922801
Phospho.ELM	MAPK8	14,71	41,67	-	-	-	91,67	84,62	0,947523
Phospho.ELM	MAPKAPK2	3,03	100	-	-	-	69,57	80	0,836332
Phospho.ELM	MAPK_group	54,9	77,78	-	-	-	82,35	75,68	0,894784
Phospho.ELM	PDK-1	42,86	85,71	-	-	-	80	95,24	0,897283
Phospho.ELM	PKA alpha	42,42	82,35	-	-	-	86,96	95,24	0,931824
Phospho.ELM	PKA_group	58,15	90,43	-	-	-	91,74	62,99	0,904783
Phospho.ELM	PKB_group	79,76	65,05	-	-	-	88,14	77,61	0,924011
Phospho.ELM	PKC alpha	19,7	72,22	-	-	-	75,56	48,57	0,825223
Phospho.ELM	PKC_group	25,21	80	-	-	-	82,02	84,39	0,900038
Phospho.ELM	PLK1	0	0	-	-	-	68,97	45,45	0,788753
Phospho.ELM	Src	0,67	5	-	-	-	74,31	65,32	0,845308
Phospho.ELM	Syk	6,67	30	-	-	-	85,71	53,57	0,878378

Comparison of best performances of the AMS 2.0 server with the current AMS version 3.0 on best results obtained on the training datasets of respective PTM types.

**Figure 2:**
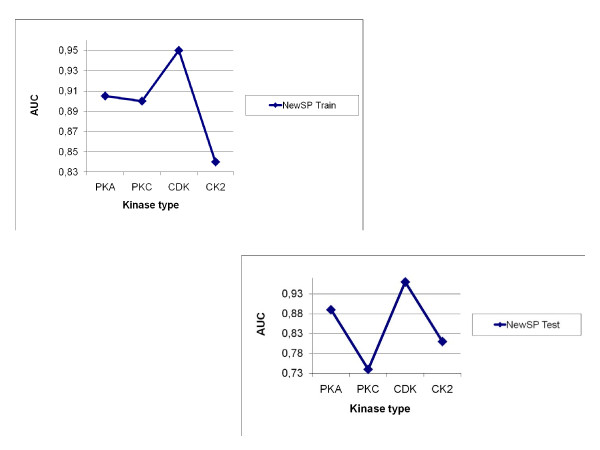
**AUC for four kinase families**. Scope of AUC values for the kinase families PKA, PKC, CDK and CK2, computed on sample train and test dataests using AMS3.

**Figure 3:**
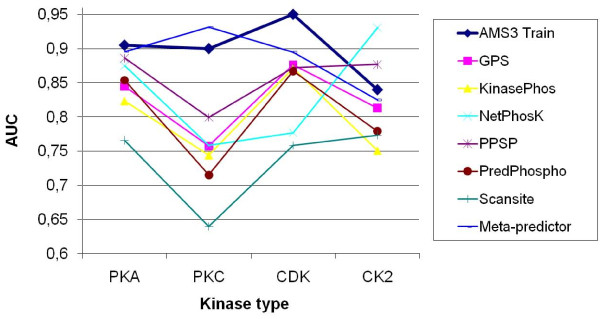
**AUC values for predictors**. Comparison of scope of AUC best values for the kinase families PKA, PKC, CDK and CK2, using AMS3, GPS, KinasePhos, NetPhosK, PPSP, PredPhospho, Scansite and Meta Predictor.

**Figure 4:**
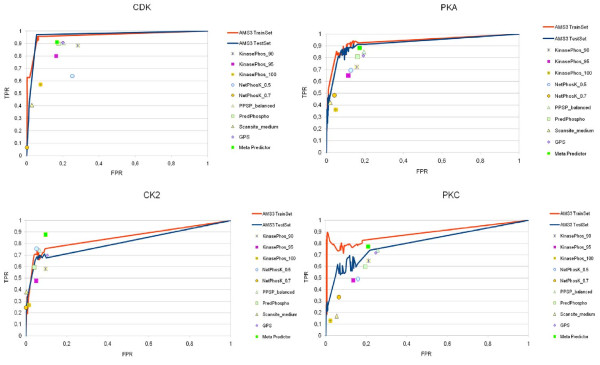
**ROC values for four kinase families**. Comparison of ROC values for the kinase families PKA, PKC, CDK and CK2, using GPS, KinasePhos, NetPhosK, PPSP, PredPhospho, Scansite and Meta Predictor with the corresponding ROC curves for training and test datasets using AMS3.

Many researchers concentrated their efforts on prediction of four major kinase families, namely CDK, CK2, PKA and PKC. To compare the current technique with the existing ones, we have conducted detailed experiments with those four kinase families from the latest Phospho.ELM dataset. Figure 2 (a-b) shows scopes of AUCs for these four kinase families for the train and test datasets of AMS3. In the current work we have compared the performance of AMS3 with the existing *state-of-the-art *prediction systems for phosphorylation sites in protein sequences. Figure 3 shows a comparative analysis of the current technique with standard predictors, namely GPS, KinasePhos, NetPhosK, PPSP, PredPhospho, Scansite and Meta Predictor. Table 3 lists the comparative performances, i.e. sensitivity, specificity and accuracy, of aforementioned prediction systems (and their variations) with the current one. In another comparison, we have plotted the ROC curves of the four kinase families Figure 4(a-d) for different runs of trainset/testset results for AMS3 and compared the same with claimed ROC values of the standard predictors.

**Table 3:** The best PTM predictors.

	Sensitivity	Specificity	Accuracy	MCC	POS
**CDK**					
GPS	0,908	0,8	0,844	0,695	294
KinasePhos_90	0,884	0,717	0,784	0,589	
KinasePhos_95	0,799	0,837	0,822	0,632	
KinasePhos_100	0,571	0,923	0,782	0,542	
KinasePhos_bitscore	0,912	0,685	0,776	0,588	
NetPhosK_0.3	1	0	0,4	N/A	
NetPhosK_0.5	0,639	0,748	0,705	0,387	
NetPhosK_0.7	0,065	0,998	0,624	0,188	
PPSP_highsens	0,983	0,075	0,438	0,128	
PPSP_balanced	0,905	0,796	0,839	0,687	
PPSP_highspec	0,054	0,982	0,611	0,1	
PredPhospho	0,898	0,823	0,853	0,708	
Scansite_low	0,667	0,884	0,797	0,571	
Scansite_medium	0,405	0,971	0,744	0,479	
Scansite_high	0,153	0,993	0,657	0,29	
Meta Predictor	0,912	0,832	0,864	0,73	
***AMS 3 (trainset)***	**0,957**	**0,94**	**0,941**		**104**
***AMS 3 (testset)***	**0,971**	**0,94**	**0,943**		
**CK2**					229
GPS	0,699	0,895	0,816	0,613	
KinasePhos_90	0,581	0,904	0,774	0,523	
KinasePhos_95	0,476	0,95	0,76	0,504	
KinasePhos_100	0,266	0,985	0,698	0,386	
KinasePhos_bitscore	0,594	0,901	0,778	0,53	
NetPhosK_0.3	0,961	0,525	0,699	0,506	
NetPhosK_0.5	0,755	0,948	0,871	0,73	
NetPhosK_0.7	0,245	1	0,698	0,403	
PPSP_highsens	0,93	0,227	0,509	0,208	
PPSP_balanced	0,742	0,933	0,857	0,7	
PPSP_highspec	0,048	1	0,619	0,171	
PredPhospho	0,594	0,959	0,813	0,616	
Scansite_low	0,576	0,983	0,82	0,64	
Scansite_medium	0,38	0,997	0,75	0,512	
Scansite_high	0,135	1	0,654	0,293	
Meta Predictor	0,878	0,904	0,893	0,779	
***AMS 3 (trainset)***	**0,733**	**0,95**	**0,921**		**248**
***AMS 3 (testset)***	**0,675**	**0,94**	**0,921**		
**PKA**					
GPS	0,817	0,809	0,812	0,618	360
KinasePhos_90	0,722	0,843	0,794	0,569	
KinasePhos_95	0,65	0,887	0,792	0,56	
KinasePhos_100	0,361	0,952	0,716	0,405	
KinasePhos_bitscore	0,775	0,804	0,792	0,573	
NetPhosK_0.3	0,878	0,724	0,786	0,59	
NetPhosK_0.5	0,694	0,874	0,802	0,583	
NetPhosK_0.7	0,483	0,959	0,769	0,525	
PPSP_highsens	0,967	0,231	0,526	0,27	
PPSP_balanced	0,85	0,806	0,823	0,645	
PPSP_highspec	0,008	0,998	0,602	0,048	
PredPhospho	0,808	0,839	0,827	0,642	
Scansite_low	0,644	0,917	0,808	0,596	
Scansite_medium	0,422	0,981	0,758	0,515	
Scansite_high	0,158	0,991	0,658	0,288	
Meta Predictor	0,883	0,828	0,85	0,699	
***AMS 3 (trainset)***	**0,917**	**0,891**	**0,896**		**345**
***AMS 3 (testset)***	**0,87**	**0,892**	**0,89**		
**PKC**					
GPS	0,718	0,753	0,739	0,466	348
KinasePhos_90	0,649	0,789	0,733	0,441	
KinasePhos_95	0,48	0,864	0,71	0,378	
KinasePhos_100	0,129	0,977	0,638	0,211	
KinasePhos_bitscore	0,687	0,722	0,708	0,404	
NetPhosK_0.3	0,716	0,695	0,703	0,403	
NetPhosK_0.5	0,491	0,841	0,701	0,358	
NetPhosK_0.7	0,333	0,935	0,694	0,348	
PPSP_highsens	0,954	0,274	0,546	0,289	
PPSP_balanced	0,741	0,743	0,743	0,477	
PPSP_highspec	0,006	1	0,602	0,059	
PredPhospho	0,598	0,805	0,722	0,412	
Scansite_low	0,411	0,866	0,684	0,315	
Scansite_medium	0,17	0,946	0,636	0,189	
Scansite_high	0,069	0,994	0,624	0,179	
Meta Predictor	0,773	0,791	0,784	0,558	
***AMS 3 (trainset)***	**0,82**	**0,98**	**0,961**		**267**
***AMS 3 (testset)***	**0,629**	**0,931**	**0,912**		

Comparison of best recognition performances of different state-of-the-art phosphorylation site prediction programs with AMS-3 for four kinase families CDK, CK2, PKA and PKC.

Among the other comparable works in this domain, the NetPhos 2.0 server predicts serine, threonine and tyrosine phosphorylation sites in eukaryotic proteins and NetPhosK 1.0 server [11] predicts kinase specific eukaryotic protein phosphoylation sites. Similar to our approach, both servers use neural network based classifiers for prediction of amino acid sequences. The performance of the NetPhosK server is reported on 5 different PTM types, i.e. PKA, PKC, CaM-II, cdc2 and CKII on independent test datasets. Table 4 shows the comparative analysis of best recognition performances of both the servers on test samples of comparable PTM types. It can be observed that our current technique also shows superior performances in all the cases under consideration. Due to variations in nomenclature and datasets, the further (unbiased) comparison with NetPhosK and other available servers could not be carried out exhaustively.

**Table 4:** NetPhosK and AMS web servers.

	NetPhosK		AMS 3.0	
	Positives	Recall	Positives	Recall
PKA	258	82	121	87.5
PKC	193	62	118	70.83
CaM-II	26	73	57	82.05
cdc2	22	37	84	88.24
CKII	85	75	248	73.3

Comparison of best recognition performances on independent test sets between the servers NetPhosK and AMS 3.0.

Average execution time for the current software is around 25 ms for 100 entries of short amino acid sequences. Each such short sequence contains nine amino acids, extracted from a complete FASTA formatted protein sequence. The experiment is conducted on a moderately powerful desktop with 1.6 GHz processor and 768 MB primary memory in Linux based operating environment.

## Discussion

The present method provides a fast and accurate system for prediction of post-translational modification sites that is capable of classifying highly complex local biological sequence features. The current design of the neural network implements three different network models for each of the PTM types by independently optimizing the network weights for optimum recall/precision/AUC values on randomly chosen test patterns.

The efficiency of classification and the prediction power of our method, estimated on training and test datasets for each PTM types using the sensitivity (recall), precision values and area under ROC curves, clearly outperforms the previously reported results. As evident in Tables 2, 3 and 4, the current technique improves the earlier versions of the AutoMotifServer (AMS 1.0, 2.0) and also outperforms the other *state-of-the-art *systems, GPS, KinasePhos, NetPhosK, PPSP, PredPhospho, Scansite and Meta Predictor, designed for kinase-specific prediction of phosphorylation sites in protein sequences. For comparison with our earlier version of AMS, the new Swiss Prot dataset could not be used because of the new nomenclature in the current version. Therefore, we partially ran the experiments with the old version of the dataset. However in the case of Phospho.ELM, the complete experimentation is done, together with the new version of the Swiss Prot dataset. Significant differences in the performance of differently optimized neural networks (for different PTM types) are observed, yet the AMS 3.0 tool integrates those heterogeneous classification schemes and it is able to boost the precision and recall values independent of a PTM type in comparison. Performances of AMS3 are also evaluated on four kinase families CDK, CK2, PKA and PKC and compared with the aforementioned predictor systems. We could observe that the performance of the current technique is comparable with the *best among the rest*. However, there are cases when the current technique fails to beat *all *other predictors. As shown in Figure 3, for the best AUC value for kinase type PKC, Meta Predictor outperforms AMS3. Similarly, in case of kinase type CK2, AMS3 comes third, below NetPhosK and PPSP. In another illustration related to ROC values, similar findings could be observed, where the ROC values of different predictors and their variations mostly come within ROC curves of AMS3 train sets and independent test sets.

There are two key reasons behind the performance boost of the current version of the AMS. Firstly, prudent choice of the feature descriptors from the AAindex database by exhaustive trial and error with different possible features. Secondly, the choice and design of the MLP base classifier. Despite popular choice of SVM in binary classification problems, we have got better accuracy with MLP by exhaustively tuning variety of *learning *parameters and experimented on a wide range of hidden neuron variations. The prudent choice of train/test ratios in positive and negative samples and the novel idea of optimizing respective networks separately on peak AUC/Recall/Precision values, also boosted the overall performance preserving the generality of the tool.

The current technique is very fast in comparison to our previously developed SVM based version of the server. As mentioned earlier, the average prediction time for 100 short amino acid sequences is estimated at around 25 ms on a standard desktop computer. In a nutshell, the MLP based pattern classifier, with independent recall/precision/AUC optimized networks, along with an effective feature descriptor is found to be more suitable to the massive prediction of post-translational modifications for whole proteomes. The availability of the precompiled, standalone version allows for high-throughput screening of large sequence datasets, the main problem that the scientific proteome community is now considering heavily.

## Conclusions

Summarizing, the AMS 3.0 tool integrates heterogeneous classification schemes for different PTM types, and it is designed to boost both the efficiency and speed in comparison with previously presented computational methods. Our fast and accurate system for prediction of post-translational modification sites in protein sequences is capable of classifying highly complex and nonlinear biological sequence patterns. We have implemented three different network models for each of the PTM types by optimizing the model for optimum recall/precision/AUC. The features for the current experiment are chosen by exhaustive experimentation on the AAindex database, based on the previously proven amino acid characteristics for prediction of *side chain interaction sites*, *secondary structure information *and related attributes. The developed *Predictor *tool, reads primary protein sequences in FASTA format and decides whether an overlapping short amino acid sequence qualifies for a potential PTM site or not, along with a probabilistic confidence for such a decision. The user also specifies the type of PTM for a specific prediction and the nature of optimization required (based on AUC area, Recall and Precision values).

The training datasets and precompiled binaries for AMS 3.0 tool are available at http://bio.icm.edu.pl/~darman/ams3 and the source code at http://code.google.com/p/automotifserver under the Apache 2.0 license scheme.

## Methods

Current dataset is extracted from the Swiss Prot Release 57.5 (dated 07-Jul-2009, consisting from 470,369 entries). All these data samples are available for free download from http://www.uniprot.org/. The dataset consists of 9 amino acid long sequence fragments centered on the post-translationally modified site used as positive instances. The negative instances were randomly selected such that they do not include experimentally verified PTM sites of any type. For each of the known thirty four (34) PTM types under consideration, separate positive and negative datasets were collected. We have also used Phospho.ELM dataset version 8.2 downloaded from http://phospho.elm.eu.org/dataset.html web site (April 2009). Phospho.ELM version 8.2 contains 4687 substrate proteins covering 2217 tyrosine, 14518 serine and 2914 threonine instances. Data from high-throughput experiments have been included.

The features for the current experiment are chosen by exhaustive optimization and search in all different hundreds (exactly 544 in the current version) of AAindex database release 9.0 http://www.genome.jp/aaindex/. Different features are considered based on their previously proven characteristics for the prediction of *side chain interaction sites *and *secondary structure information. *More specifically, features based on different side-chain interaction parameters like side-chain volume/hydrophobicity values; some typical amino acid attributes like hydration number, transfer free energy; information value for accessibility, surface tension, molecular weight etc.; and different secondary structure prediction parameters are considered for the selection of the optimum feature subset used here. We initially chose 15 different amino acid features (as shown in Table 1) from the AAindex database. Different combinations of those features descriptors are heuristically evaluated on sample train/test datasets of different representative PTM types (Phospho-PKA, Phospho-PKC, Phospho-autocatalysis and Phospho-CDC2). After exhaustive *trial and error*, ten different features are found that generate optimal recognition performance on the test datasets under consideration. Table 1 shows the list of accepted/rejected feature descriptors considered in the procedure. Possible reasons behind rejection of some apparently significant features (like ARGP820101, WARP780101 etc.) may be redundancy, or high correlation between the feature values. In other word, the rejected features failed to provide complementary/additional information to the existing feature set. The final list of accepted feature descriptors constitute a 90 dimensional feature vector for evaluation of recognition performances using an MLP based classifier on the benchmarking datasets for different PTM types under consideration. The chosen set of features can therefore be used as the best proven attributes related to post transactional modifications in amino acid sequences. Table 1 briefly lists these features (and the rejected ones) along with their AAindex accession numbers and short references.

A feed-forward artificial neural network is trained with back-propagation learning algorithm to optimize the classification accuracy between the positive and the negative samples in the randomly chosen test dataset. The optimization procedure is tuned to produce separately optimum recall, precision and the AUC area for the test dataset chosen for each of the PTM types. This is required to address specific requirements from the biologists, generating high recall/precision values for any given query sequence, using respective recall/precision optimized network setups. Also, the network setup for optimum AUC area gives balanced prediction for query sequence, resulting in moderately high both recall and precision values. The performance of each model is therefore calculated for both the training and the test datasets under consideration in three different optimization models. The classification results are generated along with a probabilistic confidence measure for such decision. More specifically, the designed neural network model generates a confidence measure *C*_*ij *_(*0 *≤ *C*_*ij *_≤ *1*) for the *i*^*th *^query sequence in the *j*^*th *^class (in this experiment, either positive or negative). This confidence measure is estimated as the normalized responses (*R*_*j*_) of respective output neurons in the output layer of the MLP. Such that, for an *i*^*th *^sequence:(5)

An MLP consists of one input layer, one output layer and one (or more) hidden or intermediate layer(s), as shown in Figure 1. The output from every neuron in a layer of the MLP is connected to all inputs of each neuron in the immediate next layer of the same as also illustrated in Figure 1. Neurons in the input layer of the MLP are dummy neurons, as they are used simply to pass on the input to the next layer just by computing an identity function.

The numbers of neurons in the input and the output layers of an MLP are chosen as respectively ninety and two for the current problem, in order to match the dimensionality of the input feature descriptor vector and the number of output classes (positives and negatives) respectively. The number of neurons in other layers and the number of layers in the MLP are determined by exhaustive trial and error method during its training. The MLP used for the present work requires supervised training. During training of an MLP weights or strengths of neuron-to-neuron connections, also called synapses, are iteratively tuned so that the MLP can respond appropriately to all training data and also to other data, not considered at the time of training. Learning and generalization abilities of an ANN are determined on the basis of how best it can respond under these two respective situations.

The MLP classifier designed for the present work is trained with the Back Propagation (BP) algorithm. The algorithm is applied to minimize the sum of the squared errors for the training samples by conducting a gradient descent search in the weight space. The number of neurons in a hidden layer is also varied during its training.

In our work we have implemented *random sub-sampling validation*, to estimate the unbiased error rate of the designed technique. This method randomly splits the dataset into training and test (validation) data. For each such split, the classifier learns to the training data, and predictive accuracy is assessed using the test data. The results are then averaged over multiple such splits. For most of the PTM types, the training and test samples for positive instances are populated in the ratio of 4:1 from all available positive samples. The number of negative samples for each type of PTM is chosen to be significantly larger than the positive samples for both the train and test datasets (the ratio of positive, negative samples is maintained as 1:5 in most cases). Random sub-sampling produces better error estimates than a single train-and-test split. The advantage of this method (over *k*-fold cross validation) is that the proportion of the training/validation split is not dependent on the number of iterations (folds). In the current work we have performed three random splits in the positive/negative datasets for each PTM. This is done so to eliminate possible bias during the training procedure in any given train/test dataset combination. The neural network for each PTM type is trained separately for the best possible AUC, the recall and precision values on the three randomly chosen *independent *test datasets. Each of these experiments, for any PTM type, is repeated at least 10 times by varying number of neurons in the hidden layer of the network. More specifically, the experiment is conducted with variation of the number of hidden neurons starting from 2 up to 20 in steps of 2. The optimum networks, giving high AUC, recall and precision values with low error rates, are saved for further consideration.

The problem of *overfitting *is addressed by optimizing each training network on *independent *test datasets, i.e., during the iterative training process the network weights that maximizes recognition accuracy on a separate test set (not the training set) is saved. Three random runs of training and test sample sets are considered for generating AUC, Sensitivity (Recall) and Precision optimized neural network models for design of the software tool. Average training and test set accuracies over these three and average over AUC and Recall accuracies are given in the in the Additional file 2.

Leave-one-out cross-validation (LOOCV) often works well for estimating generalization error for continuous error functions such as the mean squared error, but it may perform poorly for discontinuous error functions such as the number of misclassified cases (like the cases considered here). LOOCV can also run into trouble with various model-selection methods. Again, one problem is lack of continuity - a small change in the data can cause a large change in the model selected [32]. LOOCV is usually very expensive from computational point of view because of the large number of times the training process is repeated. Due to the large training data sizes for many PTM types and problem of choosing one single training model for each PTM type, LOOCV methodology is adopted in our experiments for only part of PTM types.

The developed software tool executes in two phases. In the first part, a *Sequence Generator *program reads any number of protein sequences in FASTA format, written in an input file and generates 9 amino acid long overlapping sequences for prediction. The *Predictor *program reads these short sequences and generates output. The user can specify the type of PTM for specific prediction and the nature of optimization required (based on AUC area, Recall or Precision values). The output is generated in the following format both as a binary decision (whether the sequence qualifies for a potential PTM site or not) and as a probabilistic confidence measure (*C*_*ij*_, for the *i*^*th *^query sequence in the *decision *class) for each short amino acid sequence, given as an input to the *Predictor *program. For example:

LCLYTHIGR 0 0.9853

CLYTHIGRN 0 0.9855

LYTHIGRNI 1 0.7823

YTHIGRNIY 0 0.9667

THIGRNIYY 0 0.9132

HIGRNIYYG 0 0.9763,

where, 0 and 1 signify potential negative and positive sequences respectively.

## Authors' contributions

Both authors read and approved the final version of the manuscript. SB was responsible for training of neural network, writing the MLP code, performing additional tests. DMP was involved in preparing the training dataset, designing the AMS 3.0 meta-code and its algorithm. Both authors contributed equally to the writing of manuscript.

## Supplementary Material

Additional file 1 **Supplementary materials for the publication**. Supplementary.xls contains detailed results of training and testing of AMS 3.0 tool in MS Excel file format. Click here for file

Additional file 2 **PTM predictor software with training data**. PTM_Archive.tar.gz includes the predictor software binaries together with all trained ANNs in tar.gz compressed form. Click here for file
